# A prospective randomized study comparing alfuzosin and tamsulosin in the management of patients suffering from acute urinary retention caused by benign prostatic hyperplasia

**DOI:** 10.4103/0970-1591.57917

**Published:** 2009

**Authors:** Madhu S. Agrawal, Abhishek Yadav, Himanshu Yadav, Amit K. Singh, Prashant Lavania, Richa Jaiman

**Affiliations:** Urology Division, Department of Surgery, S. N. Medical College, Agra, India

**Keywords:** Acute urinary retention, alfuzosin, BPH, trial without catheter, tamsulosin

## Abstract

**Objective:**

Prospective randomized study to compare the efficacy and safety of alfuzosin and tamsulosin in patients suffering from acute urinary retention caused by benign prostatic hyperplasia (BPH).

**Methods:**

Patients with acute urinary retention (AUR) due to BPH (total 150) were catheterized and randomized into three groups: Group A: alfuzosin 10 mg (50 patients), Group B: tamsulosin 0.4 mg (50 patients), Group C: placebo (50 patients). After three days, catheter was removed, and patients were put on trial without catheter (TWOC). Patients with successful TWOC were followed up for three months, taking into account the prostate symptom score (AUA Score), post-void residual urine volume (PVRV), and peak flow rate (PFR). ANOVA was used for statistical analysis.

**Results:**

Both group A (alfuzosin) and group B (tamsulosin) had similar results of TWOC (group A – 66%, group B – 70%), which were significantly superior than group C (placebo) – 36%. In follow up, three (9.1%) patients in group A, three (8.6%) patients in group B and eight (44.4%) patients in group C had retention of urine, requiring recatheterization. These patients were withdrawn from the study. After three months, alfuzosin- or tamsulosin-treated patients showed a significant decrease in AUA score and PVRV; and a significant increase in PFR as compared to placebo.

**Conclusions:**

TWOC was more successful in men treated with either alfuzosin or tamsulosin and the subsequent need for recatheterization was also reduced. Tamsulosin was comparable to alfuzosin in all respects, except a small but significant side effect of retrograde ejaculation.

## INTRODUCTION

Benign prostatic hyperplasia (BPH) is a highly prevalent condition in aging men, which can progress and may lead to acute urinary retention (AUR) with subsequent need for surgery.[1] Management of AUR involves immediate bladder catheterization usually followed, until recently, by prostatic surgery.[2] The greater morbidity and mortality associated with emergency surgery (within a few days after AUR), and the potential morbidity associated with prolonged catheterization (bacteriuria, fever, urosepsis), has led to an increased use of a trial without catheter (TWOC).[2]

Alpha-blockers (alpha-1 adrenoreceptor antagonists) should increase the likelihood of a successful trial without catheter (TWOC) following AUR, provided AUR is caused by increased sympathetic activity at the level of the prostatic smooth muscles.[3] Alpha-blockers (alpha(1)-adrenoreceptor antagonists) effectively reduce the symptoms associated with BPH and improve the urodynamic parameters of obstruction, without the sexual adverse events associated with the 5 alpha-reductase inhibitors.[4] They may diminish the incidence of AUR and the need for surgical intervention in symptomatic men.[4]

The advantage of tamsulosin and slow-release alfuzosin over doxazosin and terazosin in the management of AUR is that a therapeutic dose can be administered at the onset of AUR, thereby reducing the time for attempting catheter removal.[3] Third-generation alpha-blockers (alfuzosin, tamsulosin) are infrequently associated with cardiovascular side effects, in contrast to their predecessors (doxazosin, terazosin, prazosin).[5] Alpha-blocker therapy may also improve sexual functioning, with the exception of ejaculation disorders, predominantly associated with subtype selective alpha-blockers.[5]

The present study was designed to compare the efficacy of alfuzosin (10 mg daily) and modified release of tamsulosin (0.4 mg daily) with placebo in patients of BPH presenting with acute urinary retention.

## MATERIALS AND METHODS

Patients with BPH presenting to our institution with AUR (total 150), aged 48 – 90 years, were included in the study and randomized into three groups. Group A: alfuzosin 10 mg (50 patients), Group B: tamsulosin 0.4 mg (50 patients), Group C: placebo (50 patients). Only those patients experiencing their first episode of AUR who had been catheterized at our institution were included in the study. Written informed consent was obtained from all patients. Men with initial catheterization volume > 1500 ml or < 500 ml were excluded from the study. Other exclusion criteria included patients with previous prostatic surgery, known malignancy, other diseases of the bladder, retention due to medications, uremia (serum creatinine>2 mg/dl), macroscopic hematuria, urinary tract infection and hepatic impairement. The study received approval of the institutional ethical committee, and conformed to the international guidelines (The Consort Statement) for clinical trials.

**Figure 1:**
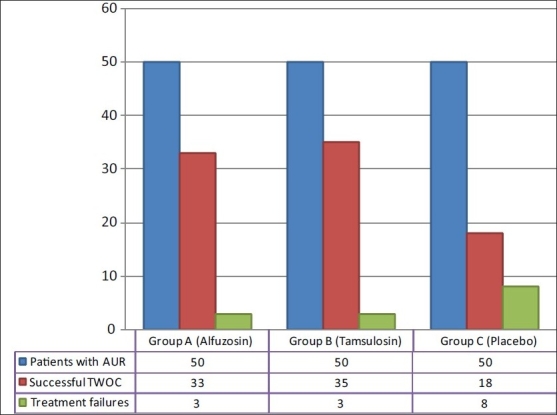
Successful TWOC in each group of patients

The study was conducted in two phases. In the first phase, patients were randomized to receive either alfuzosin 10 mg, tamsulosin 0.4 mg or placebo, with each drug administered once a day. These drugs were given after breakfast without titration. The treatment was administered for three days, after which, TWOC was given. TWOC was considered successful if the patient returned to satisfactory voiding after the removal of catheter. The primary end point of the first phase was the percentage of patients with successful TWOC [Figure 1].

In the second phase of the study, the patients with successful TWOC were allowed to continue their respective medications. The peak flow rate, post-void residual urine volume, American Urological Association (AUA) score, and drug related side effects were recorded at one week, two weeks, one month, and three months of follow up. Uroflowmetry was performed using the Phoenix V3 system (Albyn Medical 2005) to assess the peak flow rate. An assessment of the post-void residual urine volume was performed using abdominal ultrasound. The AUA symptom scores were assessed using standard questionnaires. Vital signs (heart rate, systolic and diastolic blood pressure) and spontaneously reported adverse effects were recorded at each visit. The end point of the second phase of the study was the percentage of treatment failures, defined as the need for BPH surgery or need for re-catheterization. Statistical analysis was performed using one way analysis of variance (ANOVA).

## RESULTS

The mean age of the patients in the three groups was 69.4 ± 8.8 years, 72.2 ± 8.5 years and 70.5 ± 8.0 years, respectively. The initial catheterization volume recorded was 423 ± 79.4 ml, 501.7 ± 90.7 ml and 460.3 ± 87.1 ml. The three groups were comparable in these parameters. Group A (alfuzosin) and group B (tamsulosin) had successful TWOC in 66% (33/50) and 70% (35/50) respectively, both values significantly superior in comparison to group C (placebo) – 36% (18/50) [Figure 1].

**Figure 2:**
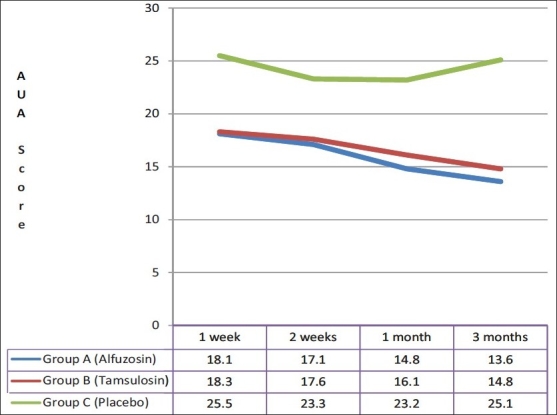
Comparison of AUA Score in follow up

**Figure 3:**
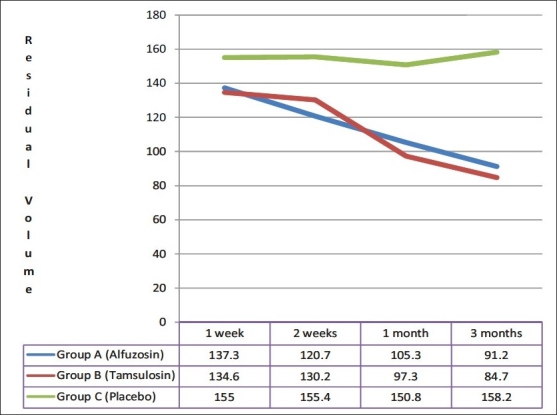
Comparison of Post void residual volume (ml) in follow up

**Figure 4:**
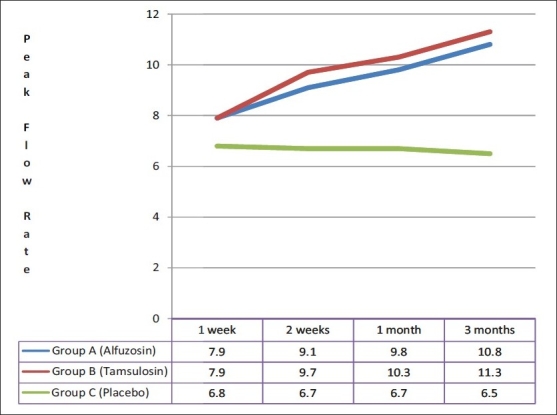
Comparison of Peak Flow Rate (ml/sec) in follow up

Among those who had a successful TWOC, there was a statistically significant difference at one week among group A, B and C patients in the AUA Score (18.1 ± 2.4, 18.3 ± 2.6 and 25.5 ± 1.9 respectively), post-void residual urine volume (137.3 ± 14.8, 134.6 ± 18.5 and 155.0 ± 27.9 ml respectively) and peak flow rate (7.9 ± 1.2, 7.9 ± 1.1 and 6.8 ± 1.5 ml/s respectively). These findings were similar among group A and B patients, but statistically significant in comparison to group C. In subsequent follow up over three months, three (9.1%) patients in group A, three (8.6%) patients in group B and eight (44.4%) patients in group C had retention of urine, requiring re-catheterization. They were withdrawn from the study and subsequently taken up for definitive treatment (transurethral resection of prostate). Of the remaining patients who were followed up for three months, those in group A and B had a significant decrease in AUA symptom score [Figure 2] and post-void residual urine volume [Figure 3]; and a significant increase in peak flow rate as compared to placebo [Figure 4]. No statistically significant differences between alfuzosin and tamsulosin treated groups were found for AUA score, post-void residual urine volume and peak flow rate [Figures 2–4].

Both alfuzosin and tamsulosin were well tolerated. The most common adverse effect with alfuzosin was dizziness (9.1% as compared to placebo 5.5%), which was statistically insignificant. In tamsulosin-treated group, headache was the most common adverse event (11.4% *vs* placebo 11.1%), which was also insignificant. Retrograde ejaculation was reported only among the patients treated with tamsulosin (11.4%). No side effects during the study required cessation of medication.

## DISCUSSION

Benign prostatic hyperplasia (BPH) is a progressive disease mainly characterized by a deterioration of symptoms over time and also the occurrence of serious outcomes such as acute urinary retention (AUR) and the need for BPH-related surgery in some patients.[6] Acute urinary retention (AUR) is a common urological emergency characterized by a sudden and painful inability to pass urine. The incidence of this complication in patients with benign prostatic hyperplasia varies widely from 0.4 to 25% in men seen in urology practices.[7] Immediate treatment consists of bladder decompression, usually by a urethral catheter. Until recently, subsequent management consisted almost exclusively of prostatic surgery within a few days (emergency surgery) or a few weeks (elective surgery) after a first AUR episode. The greater morbidity and mortality associated with emergency surgery, and the potential morbidity associated with prolonged catheterization, has led to the increased use of a trial without catheter. This involves catheter removal after one to three days, allowing the patient to void in 23-40% of cases. Surgery, if needed, can be planned at a later stage in those patients who fail TWOC.[8]

The sympathetic nervous system plays a crucial role in controlling the myogenic tone of the bladder outlet. Therefore, its activity is partly responsible for urinary outflow resistance. The alpha (1)-adrenoceptor antagonists alfuzosin, tamsulosin, doxazosin, or terazosin are able to reduce bladder outflow resistance, which leads to significant relief of LUTS (20-65%) and improvement of urinary flow (1-4.3 ml/s) in patients with symptomatic BPH.[9] Many randomized, controlled trials have provided evidence for the efficacy and tolerability of alpha (1)-adrenoceptor antagonists in LUTS/BPH, and they are the most frequently used initial treatment option for this cause of LUTS.[10]

Although all alpha-blocking compounds show similar levels of efficacy for LUTS treatment, newer agents such as alfuzosin and tamsulosin tend to demonstrate improved selectivity for the prostate and bladder. Alfuzosin (in 10 mg once daily dose) and tamsulosin (in 0.4 mg once daily dose) are better tolerated than doxazosin and terazosin due to the low risk of postural hypotension, obviating the need for dose titration.[11] Other studies have also confirmed that the benefit to risk profile of these two drugs appears to be reduced with higher doses.[12,13] Another advantage of tamsulosin and slow-release alfuzosin over doxazosin and terazosin in the management of AUR is that a therapeutic dose can be administered at the onset of AUR, thereby reducing the time for attempting catheter removal.[3] In addition, alfuzosin is the only alpha1 blocker that has demonstrated a significant decrease in post-void residual urine, a known risk factor for acute urinary retention, as well as the incidence of acute urinary retention in comparison with a placebo.[14]

The present study was undertaken to evaluate and compare the efficacy of alfuzosin and tamsulosin as compared to placebo, for treating the catheterized patients with AUR caused by BPH in order to achieve successful trial without catheter (TWOC).

In our study, TWOC was successful in 66% patients in group A (alfuzosin), which is higher than previously reported results; 55% by McNeill *et al*., 61.3% by Gopi *et al*. and 61.9% by McNeill *et al*.[15–17] Our success rate of TWOC with tamsulosin (70%) was also higher than previous observations by Lucas *et al*. and Hua *et al*., who had reported success rates of 48% and 61% respectively.[18,19]

These drugs were administered after breakfast, because food has been shown to have a clinically important effect by enhancing the bioavailability of these drugs. Both alfuzosin and tamsulosin were well tolerated. Dizziness was the most commonly observed side effect with alfuzosin, identical to that previously observed in placebo-controlled trials.[12,20,21] Alfuzosin did not cause any sexual dysfunction while tamsulosin, as reported in several previous studies, caused ejaculatory problems, which were significant as compared to placebo and alfuzosin (p<0.05).[11,22]

There have been several prospective randomized studies on the role of either alfuzosin or tamsulosin in comparison to placebo for TWOC in patients of acute urinary retention due to BPH. The present study, to the best of our knowledge, is one of the few prospective randomized trials, with head-to-head comparison between these two drugs in trial without catheter in AUR.[12] Also, most studies on TWOC are available with alfuzosin, which has been promoted as the drug of choice in this condition. Contrary to the popular perception that alfuzosin has an advantage, it seems that both drugs have a similar profile, except for a small but significant side effect of retrograde ejaculation with tamsulosin.

## CONCLUSION

TWOC in men catheterized for AUR was more successful if treated with alfuzosin or tamsulosin as compared to placebo. These patients were also less likely to need recatheterization in subsequent follow up. Among the patients who were able to void successfully, both drugs improved the prostatic symptom score, decreased the post-void residual urine volume, and improved the peak flow rate. Tamsulosin was found to be as effective as alfuzosin in all these parameters, except for sexual side effects.
